# The Helplessness Dimension of Pain Catastrophizing Mediates the Relation between PTSD Symptoms and Pain Rehabilitation Measures

**DOI:** 10.1155/2022/2112698

**Published:** 2022-11-30

**Authors:** Matthew Schumann, Julia Craner, Elizabeth Kacel, Eleshia Morrison, Keith Gascho, Judy Gebhard, Wesley Gilliam

**Affiliations:** ^1^Department of Psychiatry and Psychology, Mayo Clinic College of Medicine, Rochester, Minnesota, USA; ^2^Mary Free Bed Rehabilitation Hospital, Grand Rapids, Michigan, USA; ^3^Michigan State University College of Human Medicine, Grand Rapids, Michigan, USA; ^4^NorthShore University Health System, Chicago, Illinois, USA

## Abstract

**Background:**

Comorbid chronic pain and post-traumatic stress disorder (PTSD) complicate the treatment of both conditions. Previous research has identified pain catastrophizing as a potentially important variable contributing to the relationship between chronic pain and PTSD. However, little is known regarding how the different dimensions of pain catastrophizing—rumination, magnification, and helplessness—uniquely contribute to the relationship between PTSD symptomatology and measures of pain outcome.

**Methods:**

491 treatment seeking participants were admitted to a three-week interdisciplinary pain rehabilitation program between July 2016 and March 2020. The patients completed measures of pain severity, pain interference, pain catastrophizing, depressive symptoms, quality of life (QOL), and PTSD symptoms at pretreatment.

**Results:**

Parallel mediation analyses were conducted to evaluate the mediating effect of the Pain Catastrophizing Scale subscales on the relationship between PTSD symptomatology and pain-relevant variables. The helplessness subscale accounted for significant unique variance in the relationship between PTSD symptomatology and pain severity (*b* = 0.010, SE = 0.002, 95% CI: 0.006, 0.014), pain interference (*b* = 0.004, SE = 0.002, 95% CI: 0.001, 0.008), and mental health QOL (*b* = −0.117, SE = 0.031, 95% CI: −0.179, −0.059), while the rumination and magnification subscales had no significant influence.

**Conclusions:**

Pain catastrophizing is a multifaceted construct. These results suggest that the helplessness dimension of pain catastrophizing may be the primary target when treating patients with comorbid chronic pain and PTSD symptoms. This study represents the first to evaluate the influence of the individual dimensions of pain catastrophizing on the relationship between PTSD symptomatology and chronic pain outcome.

## 1. Introduction

Chronic pain management is often complicated by the presence of comorbid mental health conditions. A growing body of research has demonstrated that posttraumatic stress disorder (PTSD) is highly prevalent among individuals with chronic pain. Evidence suggests that approximately 20 to 37% of persons presenting for chronic pain treatment have PTSD [1] while 50 to 75% of patients who initiate care for PTSD endorse chronic pain [2, 3]. In both civilian and military populations, individuals experiencing both chronic pain and PTSD commonly self-report higher levels of pain severity and interference, greater functional impairment, and more affective distress relative to individuals with either chronic pain or PTSD alone [4, 5].

Cognitive behavioral conceptualizations of chronic pain and PTSD highlight the role of maladaptive cognitions in the development, exacerbation, maintenance, and treatment of both conditions [6]. Empiricallysupported, exposure-based treatment for PTSD, which is rooted in emotional process theory, is based on the notion that symptom reduction is facilitated when maladaptive trauma-related cognitions are challenged through systematic exposure to trauma cues in the absence of feared outcomes [7]. Similarly, conceptualizations of chronic pain theorize that an individual's catastrophic appraisals of an avoidant response to pain promote increased pain severity and functional limitations [8, 9]. In both conceptualizations, maladaptive cognitions in anticipation of or response to a feared stimulus (i.e., pain or trauma cue) can result in negative emotional responses that trigger unhelpful patterns of escape and avoidance behavior that have the unintended consequence of reinforcing unnecessary pain or fear-associated impairments.

Conceptual models have been developed that suggest that high rates of comorbidity between PTSD and chronic pain may be accounted for by either a shared vulnerability to onset and/or mutually maintaining factors following onset [6, 10]. The tendency towards interpreting ambiguous trauma- or pain-related stimuli in a catastrophic way is thought to be one factor contributing to the co-maintenance of PTSD and chronic pain [6]. Pain catastrophizing is a cognitive response style characterized by a propensity to view pain as uncontrollable, permanent, and destructive. The Pain Catastrophizing Scale (PCS) is a psychometrically validated measure commonly utilized in clinical settings to assess patients' tendencies to catastrophize in response to pain [11]. Factor analytic studies conducted on the PCS have identified three subfactors of pain catastrophizing: rumination, magnification, and helplessness [12].

The research literature has provided some support for the influence of pain catastrophizing on the relationship between PTSD symptomatology and pain outcome. For example, in a recent cross-sectional study of 203 patients with chronic pain and trauma exposure initiating care in an interdisciplinary pain rehabilitation program, pain catastrophizing (as measured by the PCS) was found to mediate the relationship between PTSD symptomatology and self-reported pain severity and interference [13]. The mediating effect of pain catastrophizing remained significant even after statistically accounting for self-reported depressive symptoms. However, tests of the reverse model (i.e., PTSD symptomatology mediating the relationship between tendency to catastrophize and self-reported pain indices) did not support PTSD symptomatology as a mediator of the pain catastrophizing and pain outcome association. Thus, the connection between PTSD symptomatology and pain outcome was primarily accounted for by pain catastrophizing. To further explore this connection, previous studies have examined the effectiveness of an interdisciplinary pain rehabilitation program in improving pain outcome (e.g., self-reported pain severity and interference) as well as PTSD symptomatology among a cohort of 83 patients with chronic pain and a provisional PTSD diagnosis [14]. Results revealed that patients self-reported significant improvements across pain outcomes and PTSD symptomatology. Notably, pre- to post-treatment reductions in pain catastrophizing fully mediated treatment-related improvements in both pain interference and PTSD symptomatology. This study provides preliminary evidence for the utility of targeting maladaptive pain-related cognitions to optimize management of both pain and PTSD symptoms in interdisciplinary pain rehabilitation programming.

In summary, accumulating evidence suggests that addressing the catastrophic appraisal patterns of individuals with PTSD and chronic pain may contribute to the effective treatment of both conditions. Further, pain catastrophizing may be an important cognitive construct to consider when examining associations between PTSD symptomatology and pain outcome. However, what is not clear from the existing literature base is how the individual facets of pain catastrophizing identified within the PCS (e.g., rumination, magnification, and helplessness) might differentially influence the identified relationship between PTSD symptomatology and pain-relevant measures, including pain severity, pain interference, depressive symptoms, and quality of life among individuals with chronic pain and trauma exposure. We contend that elucidating the impact of the individual dimensions of pain catastrophizing could further inform modifiable treatment targets among the subset of patients seeking chronic pain treatment who experience PTSD symptomatology.

The aim of this study was to evaluate the influence of the individual dimensions of pain catastrophizing on the relationship between PTSD symptomatology and chronic pain outcome. We utilized a series of mediation analyses to explore the relative contributions of the three dimensions of pain catastrophizing to the relationship between PTSD symptomatology and relevant pain measures at pretreatment among a cohort of patients with chronic pain and trauma exposure who initiated care in a tertiary-level pain rehabilitation program. Given the exploratory nature of the study, no specific hypotheses were made related to which dimensions of pain catastrophizing, if any, would mediate the relationship between PTSD symptomatology and pain-related measures.

## 2. Method

### 2.1. Participants

A total of 491 adult patients enrolled in an interdisciplinary pain rehabilitation program between the years of July 2016 and March 2020 who endorsed a Criterion A trauma (i.e., exposure to actual threatened death, serious injury, or sexual violence directly or indirectly) on the PTSD Checklist for DSM-5 (PCL-5) and completed the PCL-5 consented to participate in this study.

### 2.2. Procedures

The data were gathered from participants in a group-based, intensive, outpatient, interdisciplinary pain rehabilitation program at the Mayo Clinic. This study was approved by the institutional review board of the treatment program, which served as the site of the study. Consecutive referrals who were admitted to the Pain Rehabilitation Center at the Mayo Clinic were eligible for this study. Referrals included a diagnosed chronic pain condition in one or more anatomical sites or fibromyalgia, significant pain-related distress and disability, and consent to participate in a pain rehabilitative treatment approach. Additional inclusion criteria for this study included an endorsed trauma history and a completed PCL-5. A total of 1249 patients completed care at the Pain Rehabilitation Center during the data collection period. As noted above, 491 (39.3%) met the inclusion criteria for the study. Additional information regarding inclusion and exclusion criteria has been described previously [14]. All participants in the study consented to their data being used in future research projects upon admission into the program. As part of the admission process, participants completed a series of computerized assessment measures (see the Measures section below) and demographic information questionnaires. Previous research has found no difference between paper-and-pencil and web-based administration of commonly utilized measures [15]. All data were collected at the time of program admission (i.e., at a single time point) in this cross-sectional study.

### 2.3. Measures

#### 2.3.1. Demographics and Clinical Characteristics

Demographic and clinical characteristics were collected via a self-report questionnaire. The demographic characteristics include age, sex, race, marital status, and years of education. Primary clinical characteristics assessed include pain duration and primary pain site.

#### 2.3.2. Pain Catastrophizing

The Pain Catastrophizing Scale (PCS) was used to assess maladaptive pain cognitions [12]. The PCS is a self-report measure that consists of 13 items scored from 0 to 4. Possible scores range from 0 to 52. Higher scores indicate the individual tends to catastrophize to a greater degree. The measure consists of three validated subscales: rumination, magnification, and helplessness. The measure has been demonstrated to have robust psychometric qualities [16]. The internal consistency in the current sample for the total PCS score was high (*α* = 0.94) and was adequate to high for each of the three subscales: rumination (*α* = 0.91), magnification (*α* = 0.76), and helplessness (*α* = 0.90).

#### 2.3.3. Post-Traumatic Stress Disorder Symptoms

The PTSD Checklist for DSM-5 (PCL-5) was used to assess the presence and severity of PTSD symptoms. The PCL-5 is a 20-itemself-reported measure with responders asked to rate how bothersome their symptoms have been in the past month on a 5-point Likert scale (0 = not at all; 4 = extremely). Higher scores indicate greater symptom severity, and a score of 33 or higher is the current clinical cutoff for a provisional diagnosis of PTSD [17]. The measure has been shown to be valid and reliable in quantifying symptom severity and has demonstrated sensitivity to change [17, 18]. The internal consistency for the PCL-5 in this sample was high (*α* = 0.91).

#### 2.3.4. Pain Severity and Pain Interference

The West Haven-Yale Multidimensional Pain Inventory (WHYMPI) Pain Severity and Pain Interference subscales were used to assess the severity and suffering due to pain as well as the impact of chronic pain symptoms [19]. The pain severity subscale consists of three items, and the pain interference subscale consists of 11 items; both subscales are scored on a seven-point scale (0 = not at all; 6 = extreme). Subscale total scores reflect the average across the subscale items. The overall measure has demonstrated strong construct and convergent validity [19, 20] with adequate to excellent test-retest reliability [19, 21]. The internal consistency for the Pain Severity (*α* = 0.72) and Pain Interference (*α* = 0.88) subscales were adequate to robust for this sample of patients.

#### 2.3.5. Depressive Symptoms

The Patient Health Questionnaire-9 (PHQ-9) was used to assess depressive symptoms [22]. The PHQ-9 is a self-report measure that asks respondents to rate the presence and frequency of depressive symptoms on a four-point Likert scale (0 = not at all to 3 = nearly every day). Higher scores indicate greater symptom severity over the past two weeks. This measure has demonstrated sound psychometric properties with high internal consistency and validity [22, 23]. The PHQ-9's internal consistency was adequate for this sample (*α* = 0.83).

#### 2.3.6. Quality of Life

The physical and mental health summary scores of the 36-item Short Form Survey (SF-36) were used to assess physical and mental health quality of life. The SF-36 consists of two quality of life summary scores, physical and mental health, computed from physical and social functioning, role limitations due to physical and emotional problems, vitality, bodily pain, and general health perception domains. Items and responses are transformed into a coded value ranging from 0 to 100, with lower scores reflecting lower health-related quality of life [24]. The measure has been shown to be reliable and valid [24, 25], and further evaluation of the measure has supported the two summary scales as independent constructs of physical and mental health quality of life [26]. The internal consistency for the physical health summary (*α* = 0.84) and mental health summary (*α* = 0.82) subscales within this sample was adequate.

### 2.4. Data Analytic Strategy

To evaluate the extent to which each of the PCS subscales mediates the relationship between PTSD symptomatology and pain-related outcomes, a series of multiple mediational analyses were conducted. Specifically, the PCS subscales (e.g., rumination, magnification, and helplessness) were entered as mediators in individual models predicting the relationship between PTSD symptoms (i.e., predictor, *X*) and pain severity, pain interference, physical health-related quality of life, mental health-related quality of life, and depressive symptoms, respectively (i.e., outcome variables, *Y*). Consistent with prior research [14, 27, 28], pain severity and depressive symptoms were also included as mediators to account for their effects in the models, except where these variables were the outcome variable. Multicollinearity among mediator variables was assessed by inspecting variance inflation factors (VIFs), and results revealed no VIF exceeding 4.03 (range = 1.30–4.03), suggesting this assumption was met. Analyses were conducted with IBM SPSS version 25 using the PROCESS macro [29]. Mediation can be said to occur if the indirect effect (i.e., the relationship between the predictor and outcome variable via the mediator) is significant [30]. Significance was evaluated using bootstrap estimations for 1000 samples and 95% confidence intervals. An indirect effect is significant when the confidence interval does not cross zero.

## 3. Results

### 3.1. Demographics and Clinical Characteristics

Of the 491 patients who participated in the study, most were women (75.7%), White (91.9%), nonHispanic/Latino/a (92.7%), and married/partnered (59.5%). The average age was 48.06 (SD = 13.43) with an average years of education of 15.16 (SD = 2.89). The average duration of chronic pain was 12.21 (SD = 11.02). Participants were heterogeneous in terms of pain diagnosis, with the most common diagnoses being fibromyalgia (28.1%), low back pain (19.1%), and headache pain (14.9%). Each patient in the study had an endorsed DSM-5 Criterion A traumatic experience, and 169 (34.9%) patients scored at or above the clinical threshold for provisional PTSD with a score of 33 or greater on the PCL-V. Additional demographic information is found in Table 1, while clinic characteristics of the sample are found in Table 2.

### 3.2. Pain Severity

**Figure 1:**
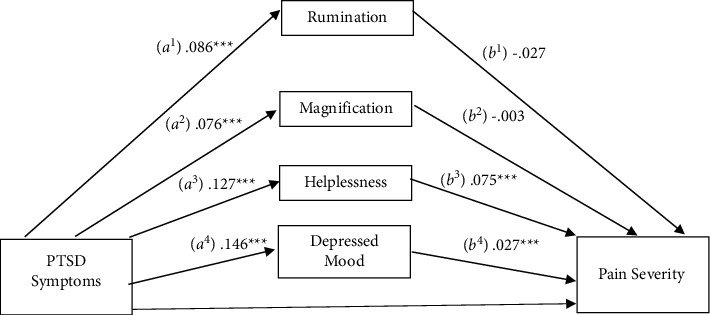
Mediation of the relationship between PTSD symptomatology and pain severity by pain catastrophizing subscales and depressed mood.

A mediational analysis was conducted to evaluate the PCS subscales as mediators in the relationship between PTSD symptoms and pain severity (Figure 1). Consistent with prior studies [13, 27, 28], depressive symptoms were included in the model to account for the variance attributable to this variable. Results suggested a significant total effect on pain severity (c pathway), *b* = 0.012, SE = 0.002, 95% CI: 0.007, 0.017. The indirect effects (ab pathways) were significant for helplessness (*b* = 0.010, SE = 0.002, 95% CI: 0.006, 0.014) and depressive symptoms (*b* = 0.004, SE = 0.001, 95% CI: 0.002, 0.007) but not for the rumination (*b* = −0.002, SE = 0.002, 95% CI: −0.005, 0.001) or magnification (*b* = −0.0002, SE = 0.002, 95% CI: −0.003, 0.003) subscales. After including these mediators, the direct effect (c' pathway) was no longer significant, *b* = 0.001, SE = 0.003, 95% CI: −0.004, 0.006, consistent with full mediation. Pairwise contrasts were conducted to compare the relative strengths of variables that had significant indirect effects. Results revealed a significant difference between depressive symptoms and helplessness, *b* = 0.006, SE = 0.003, 95% CI: 0.0003, 0.012. This indicates that the relationship between PTSD symptoms and pain severity is explained by the influence of the helplessness subscale of pain catastrophizing and depressive symptoms, and that the effect of helplessness is significantly stronger than that of depressive symptoms.

### 3.3. Pain Interference

**Figure 2:**
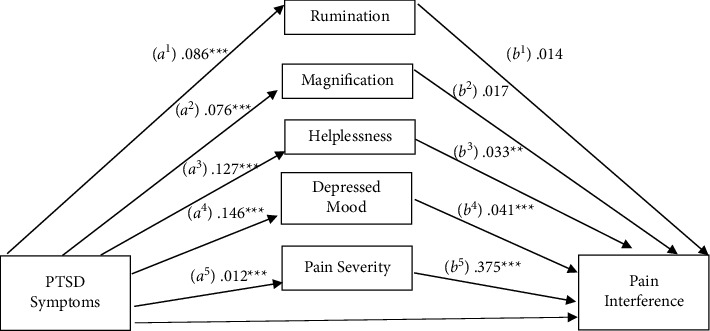
Mediation of the relationship between PTSD symptomatology and pain interference by pain catastrophizing subscales, depressed mood, and pain severity.

Next, a mediational analysis was conducted to assess the PCS subscales as a mediator in the association between PTSD symptoms and pain interference (Figure 2). Depressive symptoms and pain severity were also included as mediators to account for their influence on pain interference. Results indicated a significant total effect on pain interference (c pathway), *b* = 0.017, SE = 0.002, 95% CI: 0.013, 0.022. The indirect effects (ab pathways) were significant through helplessness (*b* = 0.004, SE = 0.002, 95% CI: 0.001, 0.008), depressive symptoms (*b* = 0.006, SE = 0.001, 95% CI: 0.004, 0.009), and pain severity (*b* = 0.005, SE = 0.001, 95% CI: 0.003, 0.007), but not through rumination (*b* = 0.001, SE = 0.001, 95% CI: −0.001, 0.004) or magnification (*b* = 0.0001, SE = 0.001, 95% CI: −0.002, 0.002). After including the mediators, the direct effect (c' pathway) was no longer significant (*b* = 0.002, SE = 0.002, 95% CI: −0.003, 0.006). Pairwise contrasts comparing the relative contribution of indirect effects indicated no significant differences. These results suggest that the relationship between PTSD symptoms and pain interference is fully mediated by helplessness, depressive symptoms, and pain severity.

### 3.4. Physical Health-Related Quality of Life

**Figure 3:**
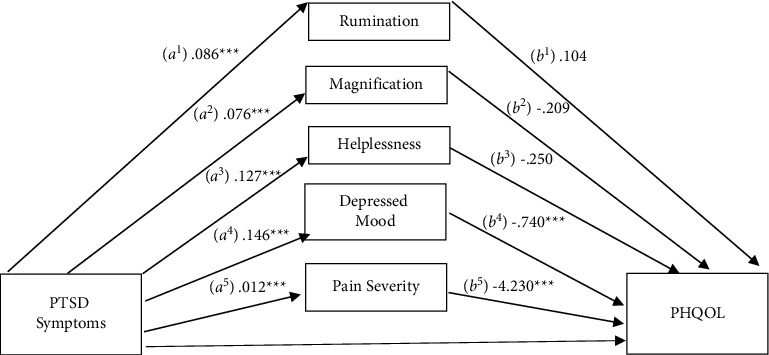
Mediation of the relationship between PTSD symptomatology and physical health-related quality of life (PHQOL) by pain catastrophizing subscales, depressed mood, and pain severity.

The PCS subscales were also evaluated as a mediator of the relationship between PTSD symptoms and physical health-related quality of life (Figure 3). As before, depressive symptoms and pain severity were included as additional mediators in the model. The total effect was significant (c pathway), *b* = −0.302, SE = 0.036, 95% CI: −0.373, −0.231. There were significant indirect effects (ab pathways) through depressive symptoms (*b* = −0.109, SE = 0.021, 95% CI: −0.152, −0.071) and pain severity (*b* = −0.051, SE = 0.015, 95% CI: −0.082, −0.025). Indirect effects were not significant for any of the PCS subscales: rumination (*b* = 0.009, SE = 0.022, 95% CI: −0.032, 0.056), magnification (*b* = −0.016, SE = 0.020, 95% CI: −0.057, 0.023), or helplessness (*b* = −0.032, SE = 0.027, 95% CI: −0.088, 0.018). After accounting for the variance attributable to the mediators, the direct effect (c' pathway) remained significant (*b* = −0.104, SE = 0.038, 95% CI: −0.178, −0.030), indicating partial mediation. The pairwise contrast comparing the indirect effects for depressive symptoms and pain severity was significant, *b* = −0.059, SE = 0.027, 95% CI: −0.110, −0.007, suggesting a stronger effect for pain severity as compared to depressive symptoms. Results of this mediational analysis indicate that depressive symptoms and pain severity partially mediate the relationship between PTSD and pain interference, while none of the PCS subscales were significant mediators.

### 3.5. Mental Health-Related Quality of Life

**Figure 4:**
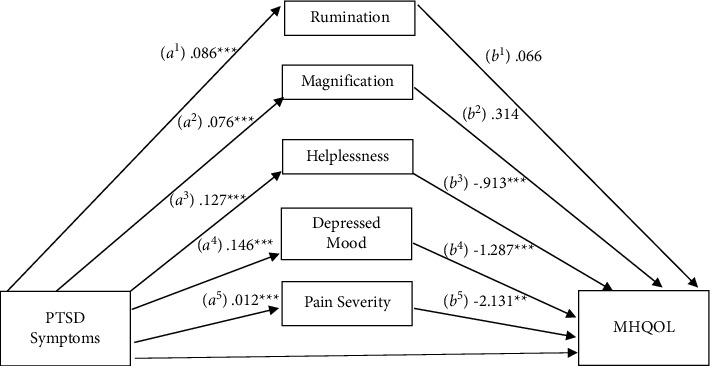
Mediation of the relationship between PTSD symptomatology and mental health-related quality of life (MHQOL) by pain catastrophizing subscales, depressed mood, and pain severity.

Next, a mediational analysis was conducted to assess the PSC subscales, depressive symptoms, and pain severity as mediators in the relationship between PTSD symptoms and mental health quality of life (Figure 4). There was a significant total effect (c pathway), *b* = −0.425, SE = 0.043, 95% CI: −0.508, −0.341. The indirect effects (ab pathways) were significant via helplessness (*b* = −0.117, SE = 0.031, 95% CI: −0.179, −0.059), depressive symptoms (*b* = −0.190, SE = 0.029, 95% CI: −0.251, −0.138), and pain severity (*b* = −0.026, SE = 0.011, 95% CI: −0.049, −0.007), but not through rumination (*b* = 0.006, SE = 0.022, 95% CI: −0.037, 0.051) or magnification (*b* = 0.024, SE = 0.027, 95% CI: −0.029, 0.077). The direct effect (c' pathway) remained significant after inclusion of the mediators, (*b* = −0.122, SE = .040, 95% CI: −0.201, −0.043), suggesting partial mediation. Pairwise comparisons indicated a significant difference between helplessness and depressive symptoms (*b* = 0.077, SE = 0.037, 95% CI: 0.005, 0.148) and depressive symptoms and pain severity (*b* = −0.004, SE = 0.002, 95% CI: −0.007, −0.0004). Overall, this analysis suggests that helplessness, depressive symptoms, and pain severity partially mediate the association between PTSD symptoms and mental health-related quality of life. The indirect effect through depressive symptoms was relatively stronger than helplessness or pain severity.

### 3.6. Depressive Symptoms

**Figure 5:**
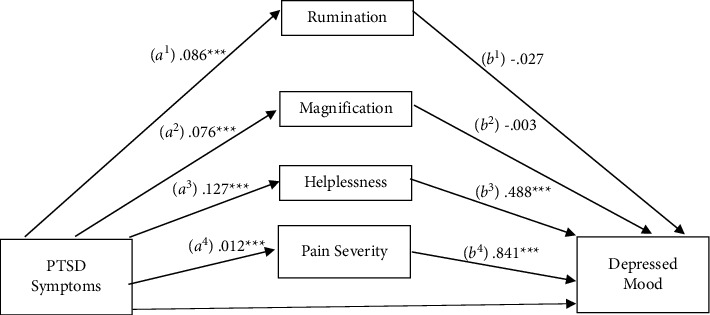
Mediation of the relationship between PTSD symptomatology and pain severity by pain catastrophizing subscales and depressed mood.

Finally, the PCS subscales were evaluated as mediators between PTSD symptoms and depressive symptoms (Figure 5). Pain severity was also included as a mediator to account for its effects. As before, the total effect on depressive symptoms was significant (c pathway): *b* = 0.146, SE = 0.013, 95% CI: 0.120, 0.172. The indirect effects were significant for helplessness (*b* = 0.062, SE = 0.012, 95% CI: 0.041, 0.086) and pain severity (*b* = 0.010, SE = 0.004, 95% CI: 0.003, 0.019), but not for rumination (*b* = −0.011, SE = 0.009, 95% CI: −0.029, 0.006) or magnification (*b* = −0.003, SE = 0.008, 95% CI: −0.018, 0.013). The direct effect (c' pathway) remained significant after accounting for the effects of the mediators, *b* = 0.088, SE = 0.013, 95% CI: 0.062, 0.114, consistent with partial mediation. The pairwise contrast comparing the indirect effects for pain severity and depression was significant *b* = 0.052, SE = 0.012, 95% CI: 0.028, 0.077, indicating a stronger effect for helplessness. Results of this analysis suggest that helplessness and pain severity partially account for the relationship between PTSD symptoms and depressive symptoms, with a stronger effect for helplessness.

## 4. Discussion

By evaluating the influence of the individual validated subscales of the Pain Catastrophizing Scale (i.e., rumination, magnification, and helplessness), we were able to elucidate a more nuanced understanding of the psychological facets of pain catastrophizing that mediate the relationship between PTSD symptoms and pain-relevant variables for individuals participating in interdisciplinary pain rehabilitation programming. The current study, which included 491 patients with chronic pain and an endorsed trauma history, revealed that helplessness significantly mediated the relationship between PTSD symptoms, pain severity, pain interference, mental health quality of life, and depressive symptoms, beyond the effects of pain severity and depressive symptoms at program admission. Pain severity and depressive symptoms were also significant mediators that helped explain the association between PTSD symptoms and the pain-relevant variables. However, when comparing the relative strengths of the mediators in these relationships, helplessness had a stronger effect on depression compared to pain severity and a stronger effect on pain compared to depression. Interestingly, the other PCS subscales (rumination and magnification) were not significant mediators in any of the analyses.

The tendency to engage in catastrophic thinking has been long thought to serve as a precursor to PTSD symptoms following the experience of stressful events [31], and individuals with chronic pain and comorbid PTSD are more likely to experience less control over pain, heightened emotional responses to pain, and higher levels of catastrophizing compared to individuals with chronic pain alone [32]. The findings are consistent with previous research suggesting pain catastrophizing serves as a potentially important cognitive variable to consider when conceptualizing the relationship between chronic pain and PTSD symptoms [13, 14, 33]. Furthermore, by utilizing within-subject mediational models, we were able to examine this model in a more granular fashion and isolate the relative contribution of each individual subfactor of the pain catastrophizing construct, with helplessness emerging as the most influential.

Helplessness due to chronic pain may be conditioned by an enduring pattern of difficulties coping with pain symptoms. Past research has shown that helplessness has a unique and significant impact on pain severity and disability [34], PTSD symptomatology [35], and the tendency to utilize maladaptive coping strategies for individuals with chronic pain and PTSD [32]. One study found that anticipatory fear and a sense of helplessness were the strongest predictors of PTSD symptoms and predicted 34% of the variance in PTSD symptomatology in individuals with trauma exposure [35]. Therefore, effective treatment for individuals with chronic pain and comorbid PTSD may be best accomplished by implementing therapeutic techniques designed to enhance distress tolerance and decrease experiential avoidance that often leads to perceptions of a lack of control over pain symptoms and anxiety. This study demonstrated that the helplessness associated with pain may be the key component of catastrophizing that perpetuates that feedback loop.

It is compelling that the magnification and ruminative dimensions of the PCS did not significantly mediate the relationship between PTSD symptomatology and pain symptoms or interference, and it is worth exploring. One explanation may be that increased magnification and rumination in response to pain and PTSD symptomatology contributes to hypervigilance to physiological symptoms and greater expression of physical and emotional distress [36] whereas magnification and rumination of pain may be typical responses of individuals with chronic pain without PTSD, and individuals with endorsed trauma may make efforts to avoid distressing pain-related thoughts or feelings associated with symptom-specific cues. For example, studies have shown that increased experiential avoidance (i.e., avoidance of coping with thoughts, emotions, or bodily sensations) predicts PTSD and increases PTSD symptoms [37–40]. This avoidance, in turn, may increase a sense of helplessness in the context of pain and PTSD symptoms, accounting for why feelings of helplessness may mediate the relationship between PTSD symptomatology and pain. A second explanation could be the chronicity of pain for participants in this study. The individuals in the study reported an average pain duration of greater than 12 years. It is possible that early in the course of the chronic pain experience, magnification and rumination were more impactful contributors to the pain and PTSD symptomatology relationship. However, as symptoms persist despite efforts to cope, it is plausible that one's perspective may shift to one of helplessness as pain becomes “the new normal” and/or pain has come to be expected with activity. These questions merit further empirical investigation, particularly using longitudinal models.

While catastrophizing may be an important process variable, assessing and addressing helplessness for individuals with chronic pain and trauma histories warrant clinical attention following the results of the current study. Education regarding helplessness or perceived control in the context of pain-related symptoms, instruction, and practice in cognitive reappraisal of pain-related threat, relaxation training, as well as systematic increases in activity, would likely yield therapeutic benefit for individuals with similar presentations to this sample. Therapeutic programs focusing on cognitive reappraisal and functional restoration have been shown to address catastrophic thinking directly or indirectly [40, 41]. Further, research has shown that reductions in catastrophizing can account for improvements in function in activity-based or interdisciplinary pain rehabilitation programming [14, 33]. Increases in activity and challenging maladaptive thoughts may increase a sense of self-efficacy in the context of pain, which may directly or indirectly target the helplessness component of pain catastrophizing.

There are important limitations when considering the outcomes of the study. First, this study utilized a cross-sectional design, and there was no treatment comparison group. Thus, longitudinal and causal relationships cannot be inferred. Second, ethnic and racial diversity was limited within this sample, limiting the ability to draw group-specific conclusions regarding differences or the generalizability of the findings. Third, tertiary-level pain rehabilitation care is accessible to a select group of people with the required resources. Therefore, the patterns of findings from this study may differ for underrepresented groups, who may have higher baseline rates of trauma. Fourth, the timing of trauma exposure in relation to PTSD symptoms and the duration of PTSD symptoms were not assessed. This information would improve the ability to examine the relationship between the chronicity of PTSD symptoms and study variables. Fifth, pain avoidance, anxiety sensitivity, and perceived injustice have been shown to be associated with chronic pain and PTSD [42, 43]; however, measures of these constructs were not included in this study. Therefore, the current study was unable to evaluate an overall conceptual model of trauma, helplessness, and other pain-relevant variables. Sixth, the number of men in the sample was relatively low (23.9%). Lastly, this study did not include measures of possible confounding variables such as emotion regulation capabilities (for example, the alexithymia construct).

In conclusion, the helplessness dimension of the PCS served as a significant mediator between posttraumatic stress disorder symptoms and pain severity, pain interference, mental health quality of life, and depressive symptoms above and beyond the contributions of other dimensions of catastrophizing and after accounting for the contributions of pain severity and depressive symptoms. These results suggest helplessness assessing and addressing maladaptive cognitions associated with helplessness for individuals with endorsed trauma histories, and chronic pain is of upmost relevance as helplessness may serve as an independent mechanism variable that can influence treatment outcome. Interdisciplinary pain rehabilitation focusing on functional restoration may be an appropriate treatment context to do so. Future studies evaluating how changes in helplessness impact chronic pain treatment and interdisciplinary pain rehabilitation outcomes are needed.

## Figures and Tables

**Table 1:** Demographic characteristics (*N* = 491).

Variable	*N* (%) or *M* ± SD
Sex	
Female	373 (75.7%)
Male	118 (23.9%)
Race	
White/Caucasian	451 (91.9%)
Black/African American	9 (1.8%)
Asian/Asian American	5 (1.0%)
Other/unknown/chose not to disclose	26 (5.3%)
Ethnicity	
Not Hispanic/Latino/a	455 (92.7%)
Hispanic/Latino/a	16 (3.3%)
Unknown/chose not to disclose	20 (4.1%)
Marital Status	
Married/partnered	292 (59.5%)
Single	137 (27.8%)
Divorced	47 (9.5%)
Widowed	8 (1.6%)
Chose not to disclose	9 (1.8%)
Age	48.06 ± 13.53
Education	15.16 ± 2.89

**Table 2:** Clinical characteristics (*N* = 491).

Variable	*N* (%) or *M* ± SD
Primary pain site	
Fibromyalgia	138 (28.1%)
Low back pain	94 (19.1%)
Headache pain	73 (14.9%)
Generalized pain	62 (12.6%)
Lower extremity	32 (6.5%)
Neck	25 (5.1%)
Upper extremity	23 (4.7%)
Abdominal	23 (4.7%)
Facial	8 (1.6%)
Pelvic/perineal/genital	8 (1.6%)
Chest wall	5 (1.0%)
Pain duration (years)	12.21 ± 11.02
PTSD symptom severity (PCL-5)	25.19 ± 18.09
Pain catastrophizing (PCS) total	26.67 ± 11.89
Rumination subscale	9.53 ± 4.27
Magnification subscale	4.66 ± 3.00
Helplessness subscale	12.48 ± 5.75
Depressed mood (PHQ-9)	13.21 ± 5.87
Pain severity (WHYMPI – PS)	4.38 ± .94
Pain interference (WHYMPI – PI)	4.70 ± 1.01
Physical health-related quality of life (SF-36 physical health summary)	29.58 ± 15.43
Mental health-related quality of life (SF-36 mental health summary)	31.62 ± 18.55

## Data Availability

The datasets generated and analysed for this study are available from the corresponding author upon request.
